# A Late Testicular Relapse in an Adult with Acute Lymphoblastic Leukemia, 5 Years after the Diagnosis and 4 Years after Allotransplant—A Rare Case

**DOI:** 10.3390/diagnostics14090875

**Published:** 2024-04-23

**Authors:** Andrei Epure, C Florin Pop, Ciprian Juravle, Florin Grosu, Carmen Naicu

**Affiliations:** 1 Department of Radiology, Sibiu, County Emergency Hospital, 540245 Sibiu, Romaniaflorin.grosu@ulbsibiu.ro (F.G.); 2 Department of Surgery, Jules Bordet Institute, Université Libre de Bruxelles, 90 Rue Meylemeersch, 1070 Brussels, Belgium; 3 Department of Histology, Lucian Blaga University of Sibiu, 550169 Sibiu, Romania

**Keywords:** CT images, ultrasound, ALL testicular relapse, sperm fluid immunophenotyping

## Abstract

Acute lymphoblastic leukemia (ALL) is a malignant disorder of lymphoid progenitor cells that affects both pediatric and adult populations. Although isolated testicular or any other organ recurrence can occur in the pediatric population, it is rare in adults. We present images for an atypical case of the late testicular recurrence of acute lymphoblastic leukemia in a 56-year-old man previously diagnosed with ALL pro-T who was in remission following polychemotherapy (GMALL 2013 protocol) and the allotransplantation of peripheral blood stem cells from a related donor. Five years later (2022), the unilateral testicular relapse of ALL was suspected by imaging and diagnosed by immunophenotyping from sperm fluid infiltrated with atypical cells with an immunophenotype concordant with that of the underlying disease (ALL T). Bone marrow aspiration and biopsy showed no evidence of systemic leukemia relapse. Testicular ablation or chemotherapy and irradiation were considered. Given the strictly testicular relapse, orchiectomy would have been useful, but given the abdominal adenopathy, a chemotherapy course with HyperCVAD Block A was first required. Testicular relapse can occur at any age, and the recognition of this is important as it may be the first manifestation of systemic relapse.

Isolated testicular relapse after acute lymphoblastic leukemia (ALL) is a rare phenomenon. This has been described mainly in the pediatric age group, with a reported incidence between 0.5% and 2% [1] and overall 5-year survival of adult patients aged 18–60 years with ALL is 20–35% [2]. There are no statistical data for the testicular relapse of ALL in adults [3,4].

With a better understanding of ALL pathology and advances in the complex diagnosis of the disease (immunophenotyping, molecular biology, and cytogenetics), the treatment and course of the disease have changed rapidly. However, relapses continue to occur, and the ideal form of treatment is still debated. We describe a case of late testicular relapse and multimodal management in an adult patient with acute lymphoblastic leukemia.

We present the case of a 56-year-old man with pro-T acute lymphoblastic leukemia (2017) in remission for whom chemotherapy, induction, and consolidation were performed under the 2013 GMALL protocol, including the prophylaxis of leptomeningeal and brain metastases with brain radiotherapy and intrathecal cytostatic administration and the consolidation of the good response with allogeneic peripheral blood stem cell transplantation from a related donor (2018) with a history of chronic viral hepatitis B. He was admitted with testicular discomfort and left testicular enlargement for one month in the urology ward. He denied testicular pain and had no associated erythema or scrotal changes; he had no fever or chills and reported no prior testicular trauma. On physical examination, the left testicle was enlarged in volume, firm, and nodular, with no cardinal signs of inflammation present; the right testicle was of normal appearance. Primary testicular carcinoma was considered. The patient presented to the attending hematologist, who together with the radiologist raised the suspicion of testicular ALL relapse given the history and keeping in mind the fact that radiotherapy and chemotherapy, as well as immunosuppressive treatment for graft-versus-host disease, could have been risk factors for primary testicular neoplastic disease. Complete blood count showed leukocytes = 7460/mm^3^, Hb = 15.3 g/dL, Ht = 43.1%, and platelets = 237,000/mm^3^. Biochemistry showed CRP = 9.6, GGT = 599, and FAS = 222.

The present report shows CT, ultrasound, and immunophenotyping images of a rare phenomenon: an isolated testicular relapse after acute lymphoblastic leukemia (ALL).

With a better understanding of ALL pathology and advances in the complex diagnosis of the disease (immunophenotyping, molecular biology, and cytogenetics), the treatment and course of the disease have changed rapidly. However, relapses continue to occur, and the ideal form of treatment is still debated.

Given the history of ALL and the patient’s symptoms, a monitoring CT scan (Figure 1a,b) was performed to assess any eventual organ relapse.

A contrast-enhanced CT scan of the thorax, abdomen, and pelvis was performed, showing an enlarged left testicle (ca. 100/60/60 mm) with increased native densities (Figure 1a), rich peripheral vascularity, discreetly iodophilic in nature, with peri-testicular fluid accumulation and epididymal contrast uptake at the level of the spermatic cord and scrotum on the left, and tissular nodules with heterogeneous iodophilia along the course of the left testicular vein to the level of the left renal hilum, in keeping with adenopathy (42/26/36 mm underlying the left renal hilum, 31/27/54 mm and 13 mm, respectively Figure 1b).

There are no statistical data for the testicular relapse of ALL in adults or CT-specific signs to be followed, and for this reason, this case is worthy to be discussed and shared.

Testicular ultrasound showed an enlarged left testicle of approx. 63/55 mm (Figure 2a), with inhomogeneous diffuse infiltrated echo structure and alternating hypoechoic areas, vascularized in Doppler and B-Flow (Figure 2b), without specific intraparenchymal masses detectable by ultrasound. Transonic peri-testicular fluid was approx. 7 mm diameter (Figure 2c).

Testicular relapse was brought up, and the modality of making a diagnosis of certainty was discussed. The ablation of the testicle would have been the first choice both diagnostically and therapeutically, but it would have delayed chemotherapy, and given the presence of adenopathy along the left testicular vein, it would have increased the risk of the systemic relapse of the disease. Testicular biopsy puncture was contraindicated, given the rich vascularity in the testicle and the fact that it was under tension.

Systemic relapse was suspected, and bone marrow (BM) aspirate was performed. An immunohistochemical examination was performed on the BM sample which did not identify atypical cells with the immunophenotype of the underlying disease (ALL T). The histopathological examination of BM appeared with mild reactive lymphoid hyperplasia, but without atypical infiltrative features. Minimal residual disease (MRD) is absent—the method’s accuracy is one pathological cell per 10^5^ cells.

A spermogram was performed which revealed the following: color, yellowish-white, slightly opalescent; viscosity, normal (at 2 h); quantity, 3.0 mL (VR: 2–6 mL); pH, 8.0 (VR: 7.2–8.0); sperm count/mL, 0.0 mil sperm cells/mL (VR: 20–200 sperm cells/mL); round cells, 5.5 mil/mL; cellularity consisting of mononuclear with morphology suggestive of blastic (lymphoblastic) cells, macrophages, polymorphonuclear neutrophils, and very rare germ cells; microbial flora was present. Flow cytometry was recommended for the accurate identification of blast-like mononuclear cells (Figure 3a–c).

Consequently, as an alternative to orchiectomy to establish the diagnosis of certainty, the immunophenotyping of spermatic fluid (Figure 3a–c) was performed; the method, according to our research and knowledge, has never been performed in Romania and, therefore, there is no standardized protocol.

While this is an unconventional method of diagnosis, without a standardized protocol, we consider it to be a valuable alternative approach in selected cases; however, the further validation of this method is required.

The immunophenotyping of the spermatic fluid (PANEL CD45, CD3s, CD3ic, CD4, CD5, CD8, and CD99) identified 36% atypical cells with the following immunophenotype: CD3ic+, CD3s−/+, CD99+, CD4−, and CD8−. The pathological product is infiltrated with atypical cells with an immunophenotype concordant with that of the underlying disease (ALL T)—analyses were performed using a Dx FLEX analyzer with 3 lasers and 13 colors (5 colors were used).

Once the diagnosis of testicular ALL relapse was confirmed, the optimal therapeutic option was discussed. Given the strictly testicular relapse, orchiectomy would have been useful [5], but given the abdominal adenopathy, a chemotherapy course with HyperCVAD [6]. Block A was required first. During the period of medullary aplasia, the patient received granulocyte growth factors, antibiotics, and antifungal treatment. The patient came out of aplasia 14 days later and was discharged.

The patient was admitted a week later for clinical, biological, and imaging evaluation and therapeutic decision; he displayed a good general condition, without hepatosplenoadenomegaly. The complete blood count performed did not change and there were no blast cells (leukemic cells) in the peripheral blood and marrow. Testicular ultrasound revealed a left testicle with dimensions of 52/28 mm, with inhomogeneous echo structure based on the presence of disseminated microcalcifications and the slight dilatation of testicular veins, with minimal adjacent edema and an increased vascular signal present in Doppler. There was a moderately dilated pampiniform plexus, moderate peri-testicular fluid collection, and free inguinal canals.

The reduction in testicular volume was attributed to the chemotherapy.

CT examination reveals the left testicle with a regressed dimensional appearance, with current dimensions of 50/40/40 mm vs. 100/60/60 mm and reduced iodophilia compared to the previous examination (Figure 4). Adenopathy along the left testicular vein appeared reduced in size with a maximum diameter of up to 20 mm.

It is also important to differentiate the testicular recurrence of ALL from primary testicular tumors such as testicular seminoma in elderly patients, as the imaging appearance is similar. Other differential diagnoses should include testicular hematoma, testicular torsion (clinical distinction), epididymal orchitis, and orchitis (overlapping sonographic features, often clinical distinction).

This case highlights the fact that testicular relapse can occur at any age, and the acknowledgement of this is important as it may be the first manifestation of systemic relapse. It raises the question of whether current surveillance protocols in ALL patients are sufficient in detecting such atypical presentations of the disease, and it emphasizes the need for early individualized monitoring strategies.

Currently, the standard protocols for the monitoring and follow-up of patients with ALL are the monthly surveillance of the complete blood count, blood smear samples, bone marrow aspirate, and minimal residual disease analysis. However, if the reoccurrence is located at the organ level only (as in the current case—localized in the testicle), by using only CBC or marrow aspirate samples with no imaging monitoring, the diagnosis can be easily missed. As the reoccurrence can occur at any organ level, it is useful to keep in mind that an individualized monitoring strategy is recommended for each patient with their own particular symptoms.

Using alternative diagnostic techniques such sperm fluid immunophenotyping can be useful in diagnosing ALL testicular relapse in selected cases, although more research is needed for a more patient-tailored approach.

## Figures and Tables

**Figure 1:**
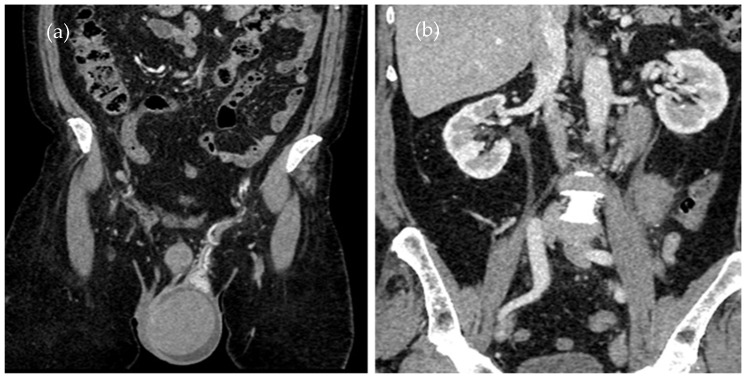
This is a figure of abdominal–pelvic contrast-enhanced CT in the coronal plane with (**a**) an enlarged left testicle and (**b**) adenopathy on the left testicular vein pathway.

**Figure 2:**
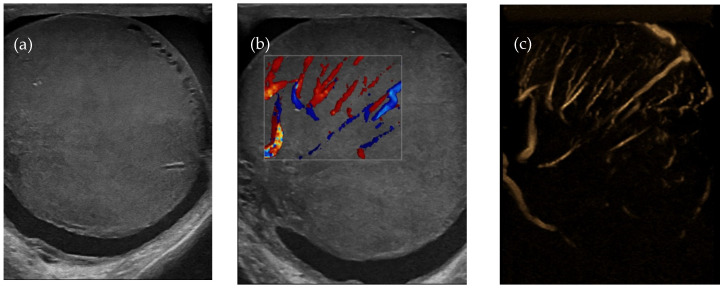
Axial ultrasound section of the left testicle: (**a**) increased volume, inhomogeneous echo structure, alternating hypoechoic areas; (**b**) vascular signal present in Doppler; (**c**) vascular signal present in B-Flow.

**Figure 3:**
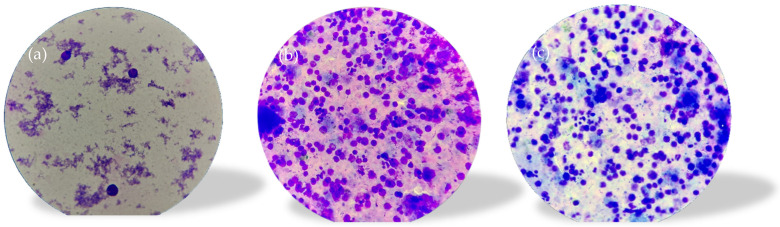
Spermogram. (**a**) Simple stretch smear technique—blasts with nucleoli; (**b**) mononuclear with morphology suggestive of blastic cells; (**c**) cytospin—concentration technique, mononuclear; microbial flora present.

**Figure 4:**
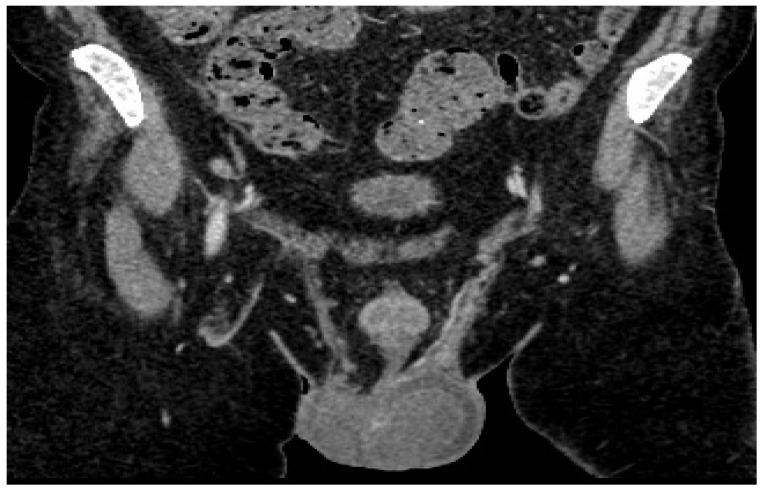
Follow-up post-chemotherapy CT of abdomen and pelvis shows left testicle and adenopathy reduced in size.

## Data Availability

For the present work, data supporting the reported results can be found in the electronic database of the Emergency Clinical County Hospital Sibiu, Romania, which provides limited access to internal users. The data are available on request from the corresponding author. The data are not publicly available due to ethical restrictions.
